# Understanding evidence and provision of services around social isolation and loneliness of military widow/ers: A scoping review

**DOI:** 10.1371/journal.pone.0293182

**Published:** 2023-11-27

**Authors:** Amy Johnson, Mary Moreland, Matthew D. Kiernan, Tracy Collins, Gemma Wilson-Menzfeld

**Affiliations:** Faculty of Health and Life Sciences, Northumbria University, Newcastle-upon-Tyne, United Kingdom; La Trobe University - Melbourne Campus: La Trobe University, AUSTRALIA

## Abstract

**Background:**

Whilst the uniqueness of loneliness and social isolation is now recognised for members of the Armed Forces Community, there is currently a lack of evidence examining these experiences within the Military Widow/er population. Therefore, this scoping review aimed to search and synthesise the current evidence base exploring experiences of loneliness and social isolation in this community.

**Method:**

Six databases were searched; ASSIA; CINAHL; ProQuest Dissertation & Theses Global; PsycArticles; Medline; Web of Science. Any article type was included if they focused on UK or international Military Widows and loneliness and social isolation. In the absence of loneliness and social isolation, related aspects were included, for example, social support.

**Results:**

A thematic synthesis was completed on the nine eligible papers, where key findings were coded and generated into four themes; Experiences of Loneliness and Social Isolation, The Uniqueness of the Military, Access to Social Support, and The Importance of Peer Support.

**Conclusions:**

Evidence supports the need for military-specific support services with peers who recognise the individuals’ unique experiences of loneliness and social isolation. None of the available evidence focused specifically on social isolation, however this was often prevalent in the results. All of the studies were carried out in the USA and Israel, with none including the views of widowers. Further evidence is required, particularly relating to a UK-context.

## Introduction

Loneliness is a subjective social and emotional experience, often considered as the inconsistency between the social relationships we have and those we wish to have [1]. In contrast, social isolation is an objective state which considers the integration of the individual in a social environment, such as the frequency of social relations and social networks [2]. Loneliness and social isolation can be experienced on their own or can be experienced simultaneously. The physical and psychological impact of loneliness, along with social isolation, is now widely recognised and includes increased risk of high blood pressure, cognitive decline, depression, and mortality [3–6]. Problematically, despite concepts of loneliness and social isolation being fundamentally different, as defined above, many accounts of loneliness or social isolation describe these simultaneously, or as one, without distinguishing between one or the other.

The unique experiences of both loneliness and social isolation in the Armed Forces community is beginning to be recognised. Most current research focusses on the experiences of loneliness [7–9] and social isolation [7–10] of military veterans. Intrinsic and extrinsic factors relating to military life can increase vulnerability to, or impact experiences of, social isolation and loneliness, including, physical health [9], and life transitions such as widowhood [11]. Compounding this, social and geographical mobility, and low social and economic capital increase the risk of experiencing adverse outcomes including social isolation [9, 10, 12].

Despite being a vulnerable population, there is however limited literature focusing on the unique experiences of social isolation and loneliness of military and war widow/ers and their support needs. The differences between the population of “War Widow/ers” and “Military Widow/ers” are complex and centre around the circumstances of death of their spouse/partner. Within the United Kingdom there are 12,348 War Widow/ers in receipt of a war widows’ pension [13], however, the actual number of War/Military Widows in the UK, not receiving a war widows’ pension, is far greater, and the true size of this population is unknown. For the purposes of this scoping review, the term Military Widow/ers will be used, and is defined as “*the recognised partner of the personnel who died in service or veteran who died following military service”* (P64) [14].

Widowhood across the general population is a complex phenomenon which can be compounded by the loss of wider familial relationships, friendships, financial hardship, and a lack of access to social support [15–17]. Furthermore, some widow/ers have restricted personal communities and limited social capital which puts them at greater risk of experiencing loneliness and social isolation [18]. However, social support is complex. Perceived social support, the quantity of supportive behaviours received [19, 20], and received social support, the satisfaction with, and availability of, social support [20, 21], are important concepts related to social capital and isolation. Particularly, there is much evidence illustrating the effect of perceived social support against physical and psychological harm following adverse life events [19, 20]. A recent systematic review shows that social support after a sudden or traumatic death reduces the severity of both symptoms of depression and Post-Traumatic Stress Disorder (PTSD) [22].

The experiences of Military Widow/ers, or War Widow/ers, may differ due to the intrinsic and extrinsic factors relating to their history of being a military spouse, with many Military Widow/ers serving in the armed forces themselves, as research carried out with military veterans has illustrated [7–10]. Furthermore, there are also significant factors relating to the notification of death that has both short-term and long-term psychological impact on Military Widows and may influence experiences of social isolation and loneliness, for example, unresolved trauma and communication of the death to family members and friends [23]. Finally, what may be especially relevant to Military Widow/ers is the potential geographical relocation if they previously lived on a military base, and this experience of losing their (Armed Forces) community in addition to the loss of their spouse [15–17]. Due to this, it is possible that the available services aiming to reduce loneliness and social isolation may be unable to account for these intrinsic and extrinsic factors.

Of the limited research in this area, McGill et al. [23, 24] explored the long-term impact of receiving the ‘knock on the door’, finding that familial relationships and friendships, as well as connections to the military, changed following bereavement. Whilst there were some challenges, the participants valued support from family and friends (particularly those from the military), and also sought out peer support groups to develop new connections. Many services exist which aim to connect individuals across the Armed Forces Community, including the population of Military and War widow/ers. A key example of this in the UK is the War Widows’ Association of Great Britain and the respective individual single service Widow Associations (Royal Navy and Royal Marine Widows’ Association, Army Widows’ Association, and the Royal Air Force Widows’ Association) which often involve one-to-one or group events and are primarily face-to-face. Recently, The War Widows’ Association of Great Britain developed a digital skills programme to connect War Widows throughout the UK in response to identifying issues of loneliness experienced by their members [25]. However, with limited evidence examining the experiences of social isolation and loneliness in this population, it is less clear what services are needed.

Despite widowhood being recognised as a key transition impacting loneliness and social isolation, there is currently a lack of research focusing specifically on Military Widow/ers experiences. As such, it is unclear what support is available to provide support for this population at both a UK and an international level. Due to the dearth of research in this area, and the limited understanding of international services available to support Military Widow/ers’ social connections, this scoping review aimed to answer the following research question:

What is the current evidence base examining experiences of social isolation and loneliness in the Military Widow/er population?

## Materials and method

A scoping review was completed to rapidly map available research focusing on service provision for loneliness and social isolation in Military Widows/ers. The scoping review followed guidance by Arksey and O’Malley [26] and Levac et al. [27]; developing research questions to guide the search strategy, locating pertinent studies, selecting studies based on inclusion criteria, ‘charting’ key data, and collating, summarising and reporting results. Ethical approval was not required for this study. The review protocol is not publicly available, however can be made available and shared upon request to the authors. A thematic synthesis was carried out in which key findings were coded in NVIVO and these codes were used to generate themes.

A database search was completed by a member of the research team (AJ) on 1^st^ November 2022 using the search terms outlined in Table 1. The search strategy was an iterative process with initial strict searches identifying little evidence in this arena and therefore, search terms purposefully remained broad. Six databases were searched (ASSIA; CINAHL; ProQuest Dissertation & Theses Global; PsycArticles; Medline; Web of Science) and were chosen based on their suitability to the research question. These databases were searched in the ‘abstract’ only, and only papers in the English language were considered.

**Table 1:** Search terms used to review published literature.

Search terms
(“armed forces” OR military OR veteran OR serving) AND (widow* OR bereav*) AND (Lonel* OR isolat* OR “social support” OR “social network”)

The eligibly criteria for inclusion in this review is presented in Table 2. As this scoping review was exploratory in nature, the eligibility criteria was intentionally broad and allowed the inclusion of grey literature (such as PhD theses) to collect any work relating to the topic of interest. As this is a scoping review, in accordance with guidelines, a critical appraisal of included papers was not conducted.

**Table 2:** Inclusion and exclusion criteria.

Inclusion Criteria	Exclusion Criteria
Focus on Military or War Widow/ers of any age, based in the UK or internationally. Articles from any year. Any article type, including theses and non-peer reviewed articles. Focus on loneliness and social isolation. In the absence of articles focusing on loneliness and social isolation, related aspects to these factors, such as social support (both confirmed as received and perceived by the military widow).	Focus on Military Veterans or currently serving personnel. Articles not focusing on loneliness, social isolation, or related factors (such as social support) experienced by Military or War Widow/ers.

**Fig 1:**
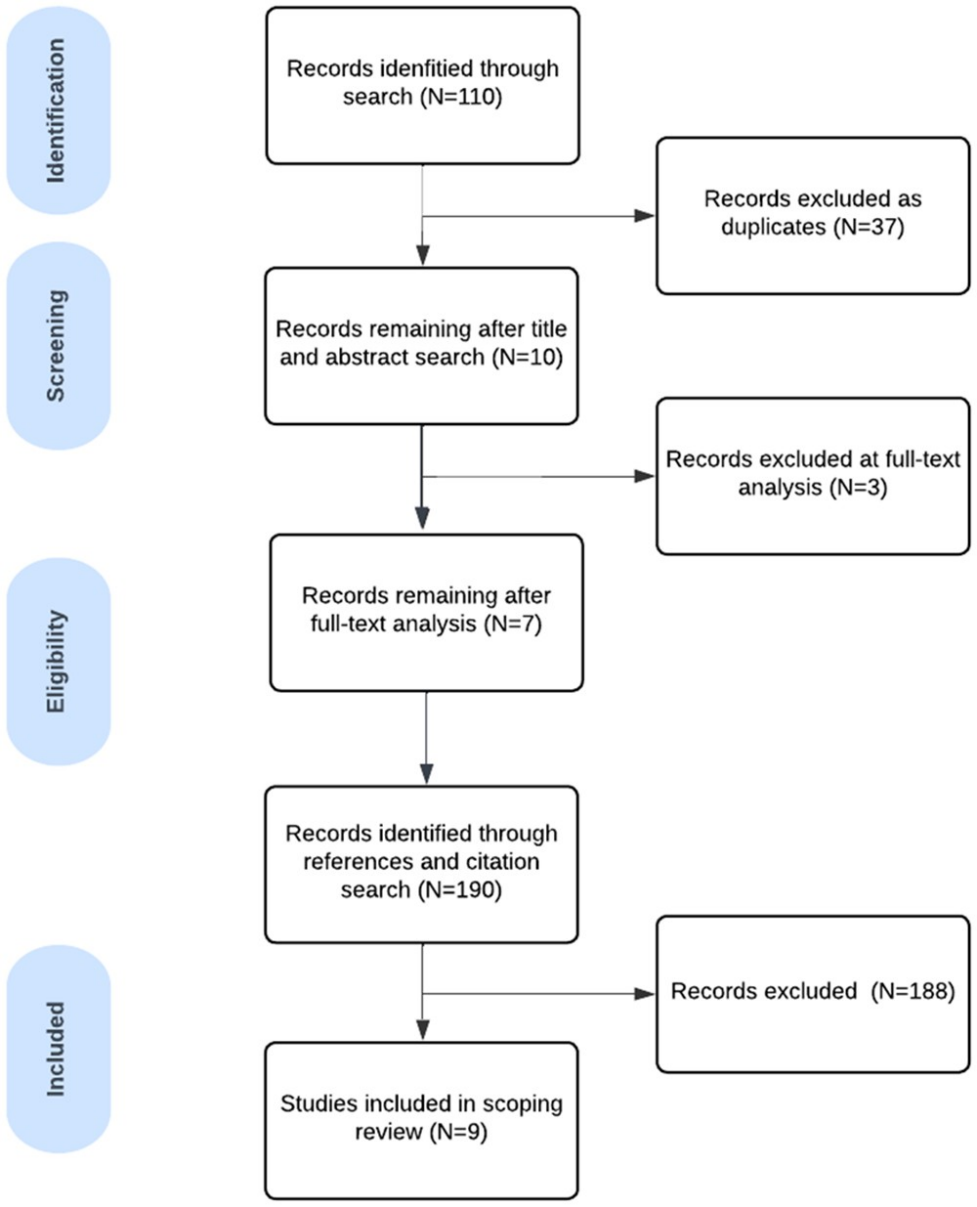
Search strategy of published literature review.

Across the six databases, 110 papers were retrieved (Fig 1). One member of the research team (AJ) led on determining study eligibility (outlined in Table 2) and regularly discussed decisions and concerns with GWM throughout the shifting and analytical process. After a title and abstract search, and removal of duplicate papers, ten papers remained. Of these, two papers were excluded as they focused on veterans who were widowed, Military Widow/ers were under-represented in the participant sample or being unable to access the full text for two articles, resulting in seven papers.

A reference and citation search was carried out between December 2022 and January 2023 and ten papers were added. Of these ten papers, eight were excluded due to primary focus being service evaluation, children of veterans and anticipatory grief; experience of grief due to diagnosis of terminal illness. Additional reasons included not focusing on loneliness and/or social isolation and under representation of Military Widow/ers in the sample. This resulted in a total of nine papers being included in this review.

First, key points from eligible articles were charted independently by members of the research team via Tables (AJ/GWM). The thematic synthesis was completed in accordance with the guidance by Thomas and Harden [28], with steps also informed by Braun and Clarke’s [29] outline for thematic analysis. Thomas and Harden [28] outline three key steps to completing a thematic synthesis: coding text, developing descriptive themes, and generating analytical themes. Supported by GWM, AJ inductively coded key findings relating to loneliness, social isolation, and social support within NVIVO before developing themes.

Both descriptive and analytical themes were generated by ‘grouping’ these initial codes by similarities and possible relationships or hierarchies. In their initial guidance, Thomas and Harden [28] describe developing analytical themes by inferring barriers and facilitators from the initial data and using these to guide the development of an intervention. Given the exploratory aim of this scoping review, and the inclusion of quantitative studies, it was difficult to follow a similar approach. As a result, the descriptive themes are presented and, where applicable, conceptual/analytical elements were extrapolated and presented within these. These are further explored within the discussion section where the themes are discussed in relation to previous findings and implications for practice are suggested.

The final output and generated themes were clarified with other members of the research team (MM and TC) due to their experiences as a peer researcher or their academic expertise in the subject area.

## Results

Eight of the papers originated in the USA and one in Israel. Four of these papers were peer-reviewed articles, one was a commentary, one was a review, and three were doctoral theses. Of the seven research outputs, four were qualitative, two were quantitative, and one was mixed methods. All the research outputs focused on female widows. Whilst Burke et al., [30] included nine male bereaved family members of veterans, based on the demographic information provided, these male participants were sons or brothers of the deceased. An overview of the paper characteristics is presented in Table 3.

**Table 3:** The published literature included within the scoping review.

Author(s)	Aim	Paper type	Design	Participants	Data collection	Country	Findings relating to loneliness and social isolation
Frye, 2012	This study aimed to understand the common aspects of grief experienced by Military Widows between 18–25, who lost their spouses during active military service	Thesis	Qualitative	Aged 18–25 Widowed within previous 12 months (Afghanistan conflict) N = 5 participants	Interviews	USA	The widow identity impacted social connection with other military spouses and led to feelings of loneliness. Peer support from other Military Widows was critical, especially from younger Military Widows. The social support required changed over time, and it was felt that more social support was needed over a longer period however, this is when social connections reduced. Feelings of loneliness were experienced by all participants, but this experience was non-linear and peaked/dipped over time.
King, Carr, & Taylor, 2021	This study aimed to examine how military service of a spouse relates to emotional adjustment following death.	Peer-reviewed paper	Quantitative	Aged 51+ Females married to male veterans and non-veterans All widowed within previous 4 years N = 428 participants; 284 veteran widows and 144 non-veteran widows	Health and Retirement Study self-report survey UCLA 3-item loneliness measure Perceived Social Support from Friends	USA	Widows of veterans experienced statistically less levels of loneliness (p < .05) consequences after bereavement compared to widows of non-veterans. This finding was due to having more resilient social support structures and mediated the relationship between widowhood and loneliness. The social support from friends declined for non-veteran widows, however, this was not the case for veteran widows for whom social support from friends was stable.
Wilson & Supiano, 2011	This study aimed to examine the grief experience of veterans’ widows.	Peer-reviewed paper	Qualitative	Aged 59–82 (mean 68.7) N = 6 participants. All veteran widows attending a bereavement group All widowed within previous 7–13 months	Interviews	USA	None of the widows in this study were in a relationship with their spouse whilst they were in the military. Social support was important for coping during bereavement however the support needs were individual and quality over quantity of social support was important.
Burke et al., 2019	This study explored the bereavement experiences of the widows of veterans of a terminal illness to identify risk factors (or predictors) of post-loss grief distress.	Peer-reviewed paper	Quantitative	Aged 29–87 (mean 58.64) N = 25 family members/friends: 12 were spouses. Pre and post death (6 to 10 weeks)	Prolonged Grief Disorder Scale Anticipatory Grief Scale NRC subscale of Brief RCOPE Inventory of Social Support Adult Attachment Scale Integration of Stressful Life Experiences Scale Neuroticism subscale of the Big Five Inventory Dyadic Adjustment Scale	USA	Lower levels of loss-related social support prior to bereavement were related to higher post-loss grief symptoms. There was a stronger relationship with bereavement-related social support and grief distress post-bereavement. Grief-related social support was included in a hierarchical regression analysis (along with relational dependence, neuroticism and meaning making) and emerged as the robust prospective predictor of grief, accounting for 15% of variation. Neuroticism, meaning making and grief-related social support post-bereavement significantly predicted complicated grief and accounted for an extra 17% of the variance in grief symptoms following bereavement.
Mitchell, 2014	This study aimed to explore the relationship between primary appraisal, secondary appraisal, coping ability, social support, stigma and bereavement. This study also aimed to determine how much these factors influenced bereavement.	Thesis	Mixed Methods	Age ranged from 25–50 years (mean = 33.48) 194 Women, aged 18+, whose spouses/partner (in the military) had died by suicide. Included 2 participants who were divorced.	Core Bereavement Items Stress Appraisal Measure Multidimensional Scale of Perceived Social Support Assessment Stigma of Suicide and Suicide Survivor Scale Coping Self Efficiency Scale 3 open questions to explain quantitative data.	USA	There was not a relationship between social support and level of bereavement. Social support was a contributor towards bereavement, along with time since death (controlled variable), primary appraisal, secondary appraisal, coping skills and stigma.
Singer, 2022	To explore Military Widow’s perspective of military widowhood and to establish the availability of and the most effective social support systems.	Thesis	Qualitative	Aged between 38–53 years 10 Military Widows bereaved from in action, training accidents, illness or suicide. Widowed between 25–48 years. Bereavement needed to occur when participants were aged between 19–50 years old to be included.	Interviews	USA	Experiences of a close Military community as a military wife which was lost following bereavement. Peer support with other widows was valuable. Military Widow Associations were a great source of support, however there was a delay joining these and lack of awareness about what was available. There was a ranking system and stigma regarding nature of death which could influence how others responded to the Military Widow. There was a variation in available resources depending on whether bereavement occurred when spouse was in service. Experiences of isolation, sometimes this was “self-imposed” as a form of protection. Mixed experience of family support following bereavement. The majority of civilian peer networks did not understand their unique experiences.
Leichtentritt, Leichtentritt, Barzilai and Pedatsur-Sukenik, 2013	To explore the lived experience of young, bereaved girlfriends of Israeli soldiers who died in service.	Peer-reviewed paper	Qualitative	15 bereaved girlfriends of Israeli soldiers (relationship length from 10 months—3years). Aged between 21–31 years. 3 of which had recently ended the relationship prior to bereavement.	Interviews	Israel	Participants described loneliness and social exclusion/lack of social support among friends and family members, who often did not recognise their loss. Support from friends and family reduced over time. Participants also described avoidance from friends who did not acknowledge the bereavement. These initially positive relationships negatively changed post bereavement. The participants chose to develop a closer relationship with the family of the deceased, which also highlighted the lack of support and consideration from others they received. Others avoided pre-existing social relationships and developed new peer networks, such as online, where the bereavement was not known.
Dooley, Carroll, Fry, Seamon-Lahiff and Bartone, 2019	Commentary on the model underpinning the Tragedy Assistance Program for Survivors (TAPS)	Commentary	n/a	n/a	n/a	USA	Whilst not specifically referring to loneliness and social isolation, peer support is an integral part of the TAPs program and plays a key role across three phases of healthy grief recovery. Access to peer support can be achieved through multiple formats; such as grief seminars, TAPS online community and peer mentorship. Accessing these support networks aims to provide ‘military survivors’ with access to a community of individuals with shared understanding and non-judgemental advice and support.
Harrington-LaMorie, Jordan, Ruocco and Cerel, 2018	To explore suicide bereavement, in particular issues relating to military suicide loss survivors, and the benefits of peer support for this population.	Review	n/a	n/a	n/a	USA	Peer support may be a form of support due to military families’ prior engagement and experiences with this. Peer support allow attendees to connect with others with similar experiences in a non-judgement environment and can reduce isolation. The TAPS Military Suicide Postvention Program is an available source of support provided to bereaved family following a bereavement. The bereaved family member often experiences isolation and loneliness following bereavement. Social support may be limited due to stigma or not knowing how to provide support, resulting in the loss of social, familial, and military support. The bereaved family member may experience blame, particularly amongst young military families, resulting in an unwillingness to seek social support.

The findings are presented as four themes: Experience of Loneliness and Social Isolation, The Uniqueness of the Military, Access to Social Support, and Importance of Peer Support.

### Experiences of loneliness and social isolation

Only Frye [31] and King et al. [32] addressed loneliness experienced by Military widows directly. King et al. [32] utilised the UCLA 3-item loneliness scale whereas Frye’s [31] study was qualitative and did not quantitively measure loneliness. Although not the primary focus, Leichtentritt et al. [33] explored the lived experiences of loneliness, isolation, and social support of bereaved girlfriends of Israeli soldiers, and Singer [34] explored experiences of Military Widows and available support networks, which included social isolation. Wilson and Supiano [35] did not directly address loneliness or social isolation, however, discuss the importance of social support during bereavement. Burke et al. [30] assessed the role of social support in post-loss grief symptoms while Mitchell [36] assessed the relationship of this with bereavement. Two articles explored the role of peer support in providing support to Military Widows [37, 38].

King et al. [32] identified that Military Widows experienced significantly lower levels of loneliness after bereavement compared to widows of non-veterans. Within Frye’s [31] study, all of the five participants experienced different levels of loneliness. These experiences of loneliness were non-linear and fluctuated over time. Leichtentritt et al. [33] noted that only marital and familial relationships are acknowledged following bereavement in Israel, one participant described their experience: “*As long as he was alive*, *I was perceived as his intimate*, *close partner*. *The moment he died*, *the relationship I had*, *which people used to acknowledge and support*, *no longer existed*!” (p812). This was reported to enhance instances of loneliness and social exclusion due to lack of public recognition of their grief.

Three articles suggested there may be elements of “self-imposed” isolation relating to withdrawing from and not wanting to burden their pre-existing support networks [31, 34, 35]. Within their review, Harrington-LaMorie [38] suggested that when bereaved from suicide, this “self-imposed” isolation could be due to shame and fear of being judged or being misunderstood.

### The uniqueness of the military

Only Wilson and Supiano [35] explored the experiences of Military Widows who became involved with their spouse following their military service. The remaining articles either focused on spouses who had experienced military life through their spouse or they did not specify this. The military community was not explored in Leichtentritt et al. [33], likely due to cultural differences and national conscription. For those who focused on this, it was clear that being a Military Wife provided individuals with access to a unique and supportive community [31, 34, 38].

Within their review, Harrington-LaMoire et al. [38] described the military as a strong “*workplace and cultural connection*” (P147) requiring support from peers. Being part of this community was seen as different to the civilian population. Military widows reported receiving limited understanding from civilian friends and family who compared their loss to civilian bereavement [31, 34]. Despite the importance of the military community, some Military Widows noted only those serving were truly part of this unique community [34].

Membership to the military community and their previous identity as a military wife was often lost or changed following bereavement [31, 34]. Participants described feeling detached from military wives, a support network they were once part of, and loss of their previous military support networks, due to avoidance and feelings of discomfort from others. One participant from Frye [31]’s study described feeling like they were “*in a whole different category now*, *a widow of a soldier rather than a wife of a soldier*” and being seen as “*an outsider like I have some kind of disease or something*” (p72). For those without family support, this could leave them completely alone. Harrington-LaMorie et al. [38] and Singer [34] suggested that the loss of these support networks could be exacerbated due to stigma regarding mental health and suicide, perceiving this as a dishonourable death. Singer [34] reported that some participants relocated nearer to family following their bereavement, due to previously existing military support networks being stationed elsewhere.

Regarding access to support, Singer [34] noted differences between Military and civilian support systems in that Military Widows could also access support specific to their spouse’s military service, such as Military Widow organisations. Often Military Widow Associations have peer support central to support provision. Dooley et al. [37] acknowledged the availability of professional mental health support in the military for those who were bereaved, however noted that there could be limited grasp of the distinctiveness of the military community and bereavement. The authors suggested that the Tragedy Assistance Programme for Survivors (TAPS) organisation overcame this issue as this was developed by a Military Widow.

### Access to social support

Burke et al. [30] found that loss-related social support was a predictor of post-grief symptomology, in particular, lower levels of social support prior to bereavement was significantly associated to greater grief symptoms post death. Within their qualitative paper, participants in Wilson and Supiano [35]’s study described a relationship between receiving perceived positive social support with improved coping following their bereavement, finding this reduced their distress. Likewise, lack of social support and increased levels of isolation was linked to higher level of distress following bereavement. In contrast, Mitchell [36] found no significant relationship been perceived social support and bereavement, despite this also being identified as an important contributor.

Military Widows’ experience of social support varied. Compared to non-veteran widows, widows of veterans reported higher levels of social support following bereavement, which was negatively correlated with change in levels of loneliness [32]. Widows who showed less distress following bereavement were positive about their social support [35]. Two participants described receiving positive support from their families following their bereavement, however others described conflict [34]. Whilst bereaved Israel girlfriends reported getting closer to their partner’s family as some form of social support, this was noted to further highlight the discrepancies in public acknowledgement of grief [33].

However, other Military Widows reported limited understanding from family and friends, particularly regarding the military aspect of bereavement [31, 33]. Participants described avoidance by family and friends, or, in some cases, feeling smothered by their constant presence [33–35]. For instance, one participant in Wilson and Supiano’s [35] study described difficulty finding some time alone; “*[family members] think it’s a crime*” and “*Sometimes you just need a breather”* (p82). Additionally, Military Widows described being blamed for their spouse’s death, being seen as a threat to their friend’s romantic relationships, being judged for failing to ‘move on’ and receiving unhelpful advice [33, 34]. Leichtentritt et al., [33] found that pre-existing relationships changed following bereavement, resulting in feelings of loneliness and limited social support. Social support was also reported to decline over time, especially following the cultural mourning period. Regarding suicide and military service, the review by Harrington-LaMorie et al. [38] suggested that the level of social support, and whether this was received, could be related to perceptions of death by suicide and associated stigma. These authors noted that previous work has also highlighted possible loss of support from the military community and the breakdown of pre-existing relationships due to these judgemental views.

Only Leichtentritt et al. [33] reported the bereaved partner sought to develop new relationships following bereavement. These new relationships, either with their significant other’s family or completely new relationships, provided instances of social support that may not be received from pre-existing social networks.

### The importance of peer support

It was clear that peer support, either through Military Widows or members of the ex-Armed Forces Community was an important source of online or face-to-face support, especially if there was limited support from friends and family [31, 33, 34, 38]. Whilst online support could be seen as valuable in communicating with other Military Widows [34] or provide the opportunity to engage with forums unrelated to their current situation [33], there remained a preference for face-to-face support [34].

Support from Military Widows, particularly from mentors further along the widowhood journey, was considered valuable due to their understanding and reducing isolation [34]. All of the participants in Fyre’s [31] study reported that peers were their primary source of support and those that were seen to match the participant’s specific situation and characteristics, such as age, were particularly valuable and reduced loneliness. In Harrington-LaMorie et al.’s [38] review, the authors discussed potential value of peer support for Military Widows following bereavement through suicide, noting there was limited evidence. They argued that connecting with individuals with shared experiences in a safe environment can provide social support and understanding, which could reduce isolation. Peer support may provide a safe environment to disclose stories, understand what is normal, and receive understanding from others.

One participant in Singer’s [34] study reported difficulty finding individuals similar to her situation. However, others noted the individuality relating to bereavement and described how their needs varied from others, for instance bereavement from suicide. In contrast, another participant argued that this was not the case, reporting bonding over the outcome of the death.

Formal processes to peer support were discussed by Dooley et al. [37] and Singer [34]. Dooley et al. [37] provided a commentary on TAPs; a service developed by a Military Widow to support bereaved families through three phases of grief. Peer support plays a pivotal role in TAPS support provision and includes access to peer supporters with personal experience of military bereavement and training to assist with grief through a variety of different mediums, including online communities, mentoring programmes, activities and 1-day events, helplines, and seminars. Dooley et al. [37] reports that TAPS can become “*like an extended family*” (p169) and allows bereaved family members to know they are not alone. Harrington-LaMorie et al. [38] reported that TAPS provide a suicide postvention program which provides access to peer-based support specific to bereavement by suicide.

All the participants in Singer’s [34] study had valuable interactions with a Military Widow organisation and credited them with providing a support system and connections with peers. The most prominent peer support service accessed by these participants appeared to be the American Widow Project. Two participants in Singer’s [34] study also engaged with TAPS, noting that, whilst this was initially beneficial, they ‘outgrew’ this. There was a preference for small groups which provided the opportunity to develop new relationships with other Military Widows. However, there was often a delay seeking this support when it would have been beneficial at the beginning of the widowhood journey.

Despite the positive implications of peer support, within their review, Harrington-LaMorie et al. [38] acknowledges challenges with this approach, such as issues with confidentiality or the possible retraumatising of attendees. These authors argue that such challenges can be overcome with clear protocols and supervision for example.

## Discussion

The present study aimed to explore the current evidence base examining experiences of social isolation and loneliness in the Military Widow/er population. Nine papers were included in this review, of which seven were research outputs, one was a commentary, and one was a review. A thematic synthesis was conducted to identify key themes included within the papers.

Four themes were generated from the included articles, the first of these being ‘Experiences of Loneliness and Social Isolation’. There were contradictory findings from these papers in terms of loneliness and the specific experiences of Military Widows compared to non-military widows. One study demonstrated that Military Widows experienced lower levels of loneliness than non-veteran widows [32] and another showed the impact of public recognition on their grief [33]. King et al. [32] explain that this finding was through more resilient perceived social support networks who were seen to provide similar levels of social support before and after spousal loss. This was in contrast to non-military bereavement in which percevied social support declined. Other studies illustrated fluctuations of loneliness over time [31] similar to that in the general population with recent evidence showing the complex, non-linear frequency of loneliness across the lifespan [39]. However, Hawkley et al. [39] evidenced universal predictors of loneliness, which did not differ with age, household income, household size, marital status, health, and frequency of socializing. Through widowhood, an individual’s experience of these universal factors will differ across their lifespan and may be dependent upon entitlement of a war widow’s pension, remarriage, and children. It is therefore as imperative to consider these factors when investigating Military Widows’ experiences of loneliness, as it is for the wider population.

The second theme ‘The Uniqueness of the Military’ highlighted the loss of the military community and lack of understanding from civilian support networks. Previous research has highlighted how intrinsic and extrinsic factors related to military service impact social isolation and/or loneliness [7–9, 12]. Whilst the literature included in this review suggested there was a loss of the military community following bereavement, McGill et al. [23, 24] found that, whilst this occurs in some cases, others valued maintaining the military connections. This is an important consideration for service provsion, in recognising that Military Widow/ers may have different experiences of loneliness and social isolation to the wider population. This strengthens the need for military-specific support servies such as the War Widows’ Association of Great Britain, and the single service widows’ assocations (Royal Navy & Royal Marines Widows’ Association; Army Widows’ Association; RAF Widows’ Association) as well as recognition of individual factors in wider service provision.

Two articles briefly reported participant’s use of online resources to seek new connections either by identifying other military widows [34], or seeking forums unrelated to their situation [33]. Dooley et al. [37] outlined the online community provided by TAPs, including blogs and message boards. There is currently little, if any, focus on using technology as a means of support for UK military widows experiencing loneliness and social isolation. The War Widows Association of Great Britain provided members with iPads and/or iPad training as a means to connect members. Results suggested that this digital technology reduced total loneliness experienced by War Widows, however had no impact on social isolation [25]. This could indicate the value of technology in developing new connections and reducing loneliness, however further work would be required as well as considering key barriers, such as digital exclusion and available skills.

The final themes generated were ‘Access to Social Support’ and ‘The Importance of Peer Support’. There were conflicting findings as to whether social support played a role in bereavement or post-grief symptomology. Burke et al. [30] found that psychosocial factors, including low levels of social support, contributed to heightened complicated grief, whereas Mitchell [36] found no significant relationship with bereavement, despite this initially being identified as a contributor. Experiences of social support also varied with some studies highlighting this as positive [32–34] while others found there was limited understanding, avoidance or blame [31, 33–35]. Within all these studies, only perceived social support was explicitly considered. These findings support the limited previous research which found that whilst bereavement could change pre-existing family and friend relationships, support provided through these networks could be invaluable [24]. However, further research is needed to directly identify the role of received and perceived social support.

Three studies examined informal peer networks and two explored formal networks for Military Widows. This review highlights the importance of peers as a valuable support mechanism. Peer support was generally valued, and was a primary source of support for some. Although this was not without its flaws, with some recognising that individual differences in the circumstances of death can impact the support that can be given by others, as well as the potential re-traumatisation. This is something that must be considered and managed within formal support services. Despite this, there appears to be limited prior research focusing on peer and/or social support for Military Widows in the UK.

One weakness in this evidence base is that none of the included studies directly investigated Military Widows’ experiences of social isolation, and this remains an international gap that needs addressing. It is also of note, that none of the included studies originate in the UK with the majority based in the USA and one in Israel.

Additionally, these papers looked at homogenous cohorts of Military Widows who were female. Although Burke et al. [30] did include male bereaved family members, these were not spousal relationships. Whilst engaging male widowers, and other seldom heard populations, in the research process has long been reported to be difficult, it is possible that the unique factors relating to the military bereaved could further impede their participation. Historically, while women have been able to serve within the UK Armed Forces for over a century, it is only since 2018 that women have been able to apply for all roles within the Armed Forces [40]. Whilst the number of women in the military has increased over time, recent statistics have shown that currently only 16,450 women are serving in the UK Regular Forces, which contribute to 11.5% of the trained and untrained strength [41]. The Ministry of Defence are aiming to increase the prevalence of women within the Armed Forces, however, there remain key concerns for female military personnel, such as bullying, sexual harassment and discrimination [40]. Whilst the number of widowers may increase in future, they will likely remain a minority compared to widows. Despite this, a key recommendation is to explore the experience of widowers, along with those from the LGBT+ population, following the bereavement of their partner who served within the Armed Forces to ensure their views are represented in developing service provisions.

Based on these points, whilst these findings can provide a useful evidence base, it could be difficult to transfer into practice within a UK setting, or to Military Widowers, due to the vast differences between the Armed Forces, and care provision internationally. Future work is needed to explore the experiences of loneliness and social isolation within UK Military Widow/ers and how to provide support. Whilst a scoping review was completed to enable the exploration of multiple study designs on a topic with possible limited focus, the absence of a quality assessment could indicate that caution should be taken with these findings. With the progression of this research topic and growing literature, there will be the ability to complete future reviews which includes a critical appraisal or quality assessment of the evidence base to further inform practice. Finally, one limitation to the studies in this review is that only one directly measured loneliness using a frequently used and internationally recognised scale (UCLA-3 scale). This is problematic as it eliminates the ability to cross-examine these cohorts and compare to the wider population.

## Conclusion

There is currently limited international evidence focussing specifically on Military Widow/ers’ experiences of social isolation or loneliness. Of this existing evidence, there is a total lack of focus on experiences of social isolation, and the evidence of experienced loneliness is contradictory but somewhat ignores the importance of universal predictors of loneliness over time. Both of these factors must be addressed in future research.

Like the research focussed on military veterans’ experiences of loneliness and social isolation, this review has shown the importance of the unique military culture for Military Widow/ers too. This is important to consider when offering services to support Military Widow/ers. Social support, and peer support, were both valuable, although not without drawbacks which must be considered within formal services.

Whilst findings can provide a useful knowledge base, differences in culture means these can be difficult to transfer to a UK setting. The focus on female participants can mean that these findings may not be transferable to Military Widowers. Previous research highlights the role of identity with social connections, and the value of peer and social support on loneliness and coping, however it is important to consider transferability to a UK setting.

There is a major gap in knowledge regarding the impact and experiences of Military Widows accessing formal support to reduce experiences of loneliness and/or social isolation. Future research could further explore this area and refine support for Military Widows/ers experiencing loneliness and social isolation.

## Supporting information

S1 Checklist Preferred Reporting Items for Systematic reviews and Meta-Analyses extension for Scoping Reviews (PRISMA-ScR) checklist. (PDF)
